# PI3K/AKT signaling activation by roflumilast ameliorates rotenone-induced Parkinson’s disease in rats

**DOI:** 10.1007/s10787-023-01305-x

**Published:** 2023-08-04

**Authors:** Heba A. Farid, Rabab H. Sayed, Marwa El-Sayed El-Shamarka, Omar M. E. Abdel-Salam, Nesrine S. El Sayed

**Affiliations:** 1 Department of Narcotics, Ergogenic Aids and Poisons, National Research Centre, Cairo, Egypt; 2 Department of Pharmacology and Toxicology, Faculty of Pharmacy, Cairo University, Kasr El Aini St., Cairo, 11562 Egypt

**Keywords:** Apoptosis, Inflammation, Neuroprotection, Parkinson’s disease, PDE4 inhibition

## Abstract

Parkinson’s disease (PD) is the second most common progressive age-related neurodegenerative disorder. Paramount evidence shed light on the role of PI3K/AKT signaling activation in the treatment of neurodegenerative disorders. PI3K/AKT signaling can be activated via cAMP-dependent pathways achieved by phosphodiesterase 4 (PDE4) inhibition. Roflumilast is a well-known PDE4 inhibitor that is currently used in the treatment of chronic obstructive pulmonary disease. Furthermore, roflumilast has been proposed as a favorable candidate for the treatment of neurological disorders. The current study aimed to unravel the neuroprotective role of roflumilast in the rotenone model of PD in rats. Ninety male rats were allocated into six groups as follows: control, rotenone (1.5 mg/kg/48 h, s.c.), L-dopa (22.5 mg/kg, p.o), and roflumilast (0.2, 0.4 or 0.8 mg/kg, p.o). All treatments were administrated for 21 days 1 h after rotenone injection. Rats treated with roflumilast showed an improvement in motor activity and coordination as well as preservation of dopaminergic neurons in the striatum. Moreover, roflumilast increased cAMP level and activated the PI3K/AKT axis via stimulation of CREB/BDNF/TrkB and SIRT1/PTP1B/IGF1 signaling cascades. Roflumilast also caused an upsurge in mTOR and Nrf2, halted GSK-3β and NF-ĸB, and suppressed FoxO1 and caspase-3. Our study revealed that roflumilast exerted neuroprotective effects in rotenone-induced neurotoxicity in rats. These neuroprotective effects were mediated via the crosstalk between CREB/BDNF/TrkB and SIRT1/PTP1B/IGF1 signaling pathways which activates PI3K/AKT trajectory. Therefore, PDE4 inhibition is likely to offer a reliable persuasive avenue in curing PD via PI3K/AKT signaling activation.

**Graphical Abstract:**

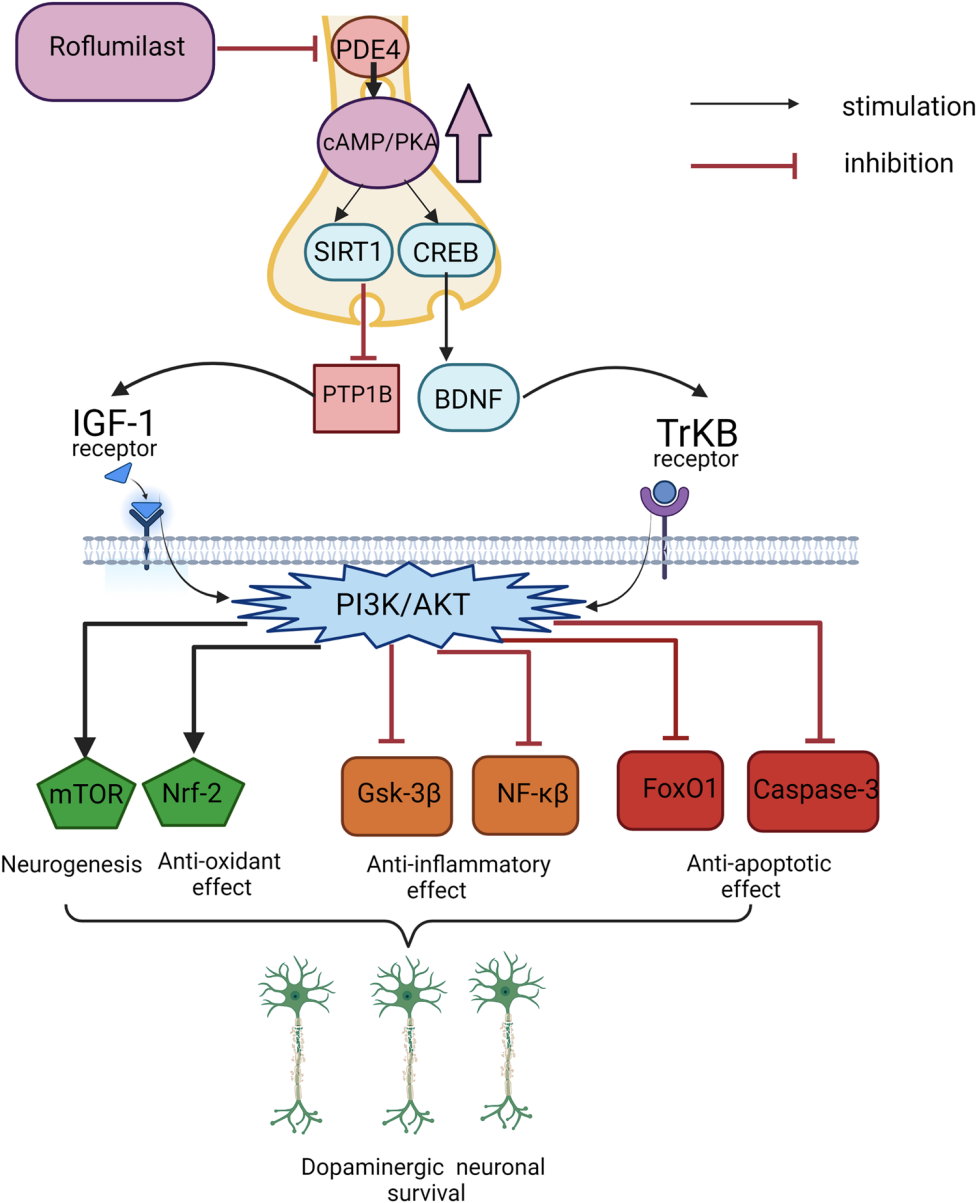

## Introduction

Parkinson’s disease (PD) is the second most common progressive age-related neurodegenerative disorder **(**Aarsland et al. 2021; Tryphena et al. 2023**)**. The most distinctive symptoms of PD are motor deficits, such as bradykinesia, tremors, and freezing gait disturbances **(**Tarakad and Jankovic 2017**)**. These motor symptoms result from persistent loss of striatal dopaminergic (DAergic) neurons in the substantia nigra pars compacta (SNpc) leading to dopamine (DA) deficiency in the striatum (Chakrabarti and Bisaglia 2023). The degeneration of dopaminergic neurons is driven by Lewy bodies, which are formed from misfolded α-synuclein (α-Syn) protein aggregates (Iarkov et al. 2021). Oxidative stress, mitochondrial dysfunction, neuroinflammation, and apoptosis are regarded to be the main mechanisms exacerbating the deleterious potential of α-Syn predisposing dopaminergic neurons to further demise (Hassanzadeh and Rahimmi 2018; Musgrove et al. 2019; Dionísio et al. 2021).

The currently approved pharmacological treatment for PD includes levodopa, dopaminergic receptor agonists, and anticholinergic drugs (Rezak 2007). However, they provide symptomatic relief only without halting the disease progression. Furthermore, these drugs produce significant adverse effects such as levodopa-induced dyskinesia and wearing-off phenomenon (Armstrong and Okun 2020). Therefore, research is diverted toward investigating novel therapeutic approaches in PD treatment (Keighron et al. 2023).

Paramount evidence shed light on the activation of phosphoinositide 3-kinase (PI3K)/protein kinase B (AKT) signaling pathway and its worthwhile neuroprotective role in PD (Yao et al. 2022; Li et al. 2023b). PI3K/AKT cascading axis, via its impact on a plethora of proteins, has been established to be one of the most crucial pathways capable of ameliorating neuronal survival, improving neurogenesis, and repressing apoptosis induced by neurotoxins in PD models (Zheng et al. 2021a, b; Khezri and Ghasemnejad-Berenji 2022; Wang et al. 2022). PI3K/AKT is activated after the binding of diverse neurotrophic factors to their membrane receptors including brain-derived neurotrophic factor (BDNF)/tropomyosin receptor kinase B (TrKB) (Jin et al. 2022; Gendy et al. 2023) and silent information regulator type 1 (SIRT1)/insulin growth factor 1 (IGF1) cascading axes (Yang et al. 2018; Flores et al. 2023; Arjunan et al. 2023).

Noteworthy, BDNF and SIRT1 cascading axes can be provoked with the aid of enhancing cyclic adenosine monophosphate (cAMP) magnitude (Bhat et al. 2020; Dong et al. 2021). The cAMP level is proven to be elevated via phosphodiesterase 4 (PDE4) inhibition (Kelly 2018). Intriguingly, PDE4 inhibition was implicated as a reliable target in ameliorating PD (Nthenge-Ngumbau and Mohanakumar 2018; Roy et al. 2023) as well as promoting proteasomal degradation of α-Syn deposits in PD (Desouky et al. 2023).

Roflumilast is a selective PDE4 inhibitor that is approved by the FDA for the treatment of severe chronic obstructive pulmonary disease (COPD) (Janjua et al. 2020). Roflumilast is established to be brain penetrant targeting PDE4 sites in the cortico-striatal-thalamic circuitry including the nigral area (Vanmierlo et al. 2016; Heckman et al. 2018). Furthermore, roflumilast has been proposed as a favorable candidate for the treatment of neurological disorders such as Alzheimer’s disease, cerebral ischemia, sleep deprivation-induced cognitive deficits, and depression via improving neuroinflammation, memory, and cognition (Wang et al. 2020a; Vilhena et al. 2021; Bhat et al. 2022; Zaki et al. 2023). Recently, Desouky et al. (2023) illustrated that roflumilast is capable of prompting proteasomal degradation of detrimental α-Syn deposits in PD animal model. Also, Essam and Kandil (2023) reported that roflumilast halts the progression of rotenone-induced PD in rats via activation of cAMP-PKA signaling pathways. Taken together, the current study aimed to unravel the plausible neuroprotective role of roflumilast in the rotenone model of PD in rats by focusing on targeting PI3K/AKT signaling cascade.

## Material and methods

### Chemicals and drugs

Rotenone and roflumilast were purchased from Sigma-Aldrich (St. Louis, MO, USA), while dimethyl sulfoxide (DMSO) and carboxymethylcellulose (CMC) were obtained from Merck (Darmstadt, Germany) and Santa Cruz Biotechnology (Santa Cruz, CA, USA), respectively. Levodopa was acquired from Merck and Co. Inc. (New Jersey, USA). All used chemicals were of the highest purity and analytical grade. Rotenone was prepared in 1% DMSO, while roflumilast was suspended in 1% CMC.

### Animals

Male Wistar rats weighing 200–250 g were obtained from the National Scientific Research Centre (Giza, Egypt). The animals were grouped before the experiment and housed under controlled environmental conditions of constant temperature (25 ± 2 °C), humidity (60 ± 10%), and a 12/12-h light/dark cycle with free access to standard chow diet and water. The investigation compiled with the Guide for the Care and Use of Laboratory Animals published by the US National Institutes of Health (NIH Publication No. 85–23, revised 2011) and was performed in agreement with ethical procedures approved by the Ethics Committee of Faculty of Pharmacy, Cairo University (Permit Number: PT 2481).

## Experimental design

**Fig. 1:**
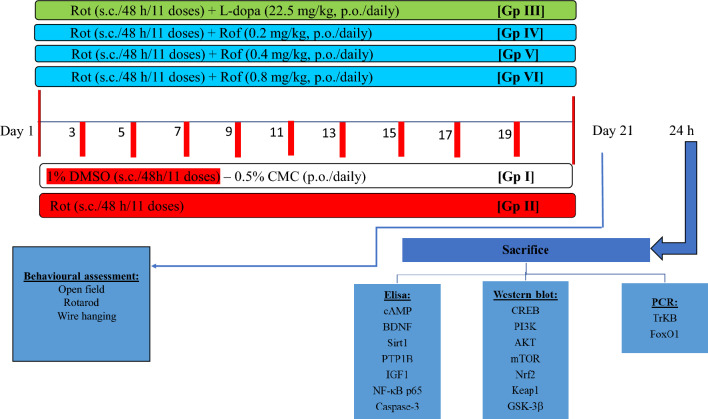
Experimental design. *DMSO* dimethylsulfoxide, *CMC* carboxymethylcellulose, *Rot* rotenone, *Rof* roflumilast

As shown in Fig. 1, a total of ninety rats were randomly divided into 6 groups, each of 15 animals. Group I received 1% CMC (2.5 ml/kg, p.o.) daily in addition to 11 injections of 1% DMSO (0.2 ml/kg, s. c.) on rotenone corresponding days and served as a control group. Group II was treated with 11 injections of rotenone (1.5 mg/kg, s.c) dissolved in 1% DMSO every other day for 21 days (Mansour et al. 2018). Group III was treated daily with L-dopa dissolved in saline solution (22.5 mg/kg, p.o) 1 h after rotenone injection (Ahmed-Farid et al. 2021) for 21 days. Roflumilast (0.2, 0.4, or 0.8 mg/kg, p.o., suspended in 0.5% CMC) was administered daily for 21 days 1 h after rotenone injection to rats of Group IV, V, and VI, respectively (Feng et al. 2019). Twenty-four hours after the last rotenone injection, rats were screened for motor performance using the open field, rotarod, and wire hanging tests. These tests were set in order from the least stressful to the most stressful and were all conducted during the animals’ light cycle to decrease circadian variability.

After behavioral assessments, animals were sacrificed by cervical dislocation under light anesthesia. Brains were rapidly excised and washed with ice-cold saline. Dissection of each brain was conducted on an ice-cold glass plate for separation of striata. Animals in each group were further divided into three sets. In the first set (*n* = 6), both striata were homogenized in 10% (w/v) saline and were used for assessments via the enzyme-linked immunosorbent (ELISA) technique. The striata of the second set (*n* = 6) were designated for Western blot and qRT-PCR analysis. Brains of the last set (*n* = 3) were fixed in 10% (v/v) formalin for 24 h to execute histopathological staining with hematoxylin and eosin (H&E). Nuclear extraction was done for proteins whose active forms are mainly expressed in the nucleus including NF-κB and Nrf2.

## Behavioral assessment

### Open field test

The open field test was carried out using a square wooden box measuring 80 × 80 × 40 cm with red walls and a black smooth polished floor divided by white lines into 16 equal squares. Each rat was placed gently in the central area of the open field and allowed to freely explore the area for 3 min. The floor and walls were cleaned with 10% alcohol after testing each rat to eliminate possible bias due to odors left by previous rats. A video camera was fixed on the top of the box to record the movement and behavior of rats for later offline analysis. Ambulation frequency (number of squares traversed by the animal) was recorded (Tatem et al. 2014).

### Rotarod test

Evaluation of motor coordination and balance using a rotarod apparatus (3 cm diameter, 90 cm height, and 10 rpm) was performed. Rats were trained for 3 successive days to remain on the stationary and rotating rod (three sessions, 5 min each). Before the sacrifice and after accomplishing the open field test, animals were allowed to move over the rotarod for 5 min, and their falling time was recorded (Jones and Roberts 1968).

### Wire hanging test

To evaluate motor strength and muscular rigidity, rats were suspended by their forelimbs from a steel rod (20 cm long and 0.25 cm in diameter) located 20 cm above the bench (Model 47200, Ugo Basile, Comerio, Italy). The forelimb grip strength was assessed using a grip strength meter. Each rat was placed horizontally over a base plate facing a triangle bar and then dragged steadily by its tail away from the bar upon grasping it until its grip was lost. The latency time to fall was recorded (Massicotte et al. 2015).

## Biochemical parameters

### ELISA

The striatal contents of BDNF, nuclear factor erythroid related factor 2 (Nrf2), nuclear factor kappa beta (NF-κB p65), cAMP, SIRT1, and protein tyrosine phosphatase 1B (PTP1B) were estimated according to the manufacturer’s prescripts provided by rat ELISA kits (Cat. # MBS824814, MBS012148, MBS015549, MBS2700004, MBS2600246, MBS3809151 MyBioSource, CA, USA, respectively). Likewise, IGF1 and caspase-3 were assayed using rat ELISA assay kits (Cat. # CSB-E04582r, CSB-E08857r Cusabio, Wuhan, China, respectively) using BioTek Elisa Reader ELx808. The procedures were performed following the manufacturers’ directions. The results were expressed as pg/mg protein for BDNF, NF-ĸB p65, cAMP, and Nrf2; ng/mg protein for SIRT1, IGF1, and caspase-3 levels; and as µg/mg protein for PTP1B; where the protein content was determined using Bradford method (Bradford 1976).

### Western blot analysis

The protein expression of striatal cAMP response element-binding protein (CREB), AKT, mammalian target of rapamycin (mTOR), and glycogen synthase kinase-3 beta (GSK-3β) proteins was assessed using the Western blot analysis. After protein solutions were extracted from striatal tissues, equal amounts of proteins were loaded onto a sodium dodecyl sulfate–polyacrylamide gel electrophoresis, which allows the separation of proteins according to their molecular weight. Subsequently, the samples were electro-transferred onto nitrocellulose membranes (Amersham Bioscience, Piscataway, NJ, USA) using a semidry transfer apparatus (Bio-Rad, Hercules, CA, USA). These membranes were blocked with 5% non-fat dry milk in Tris-buffered saline with 0.05% Tween-20 (TBST) for 1 h at room temperature, and incubated overnight at 4 °C on a roller shaker with antibody against rat anti p-CREB (ser133) (1:1000, Catalog No. # MA5-11192), anti p-PI3K (ser110α) (1:500, Catalog No. # PA5-87398), anti p-AKT1 (ser473) (1:500, Catalog No. # PA5-85513), anti p-mTOR (ser2448) (1:500, Catalog No. #MA5-35832), anti-Nrf2 (1:500, Cat # PA5-27882), anti-Keap1 (1:500, Cat #PA5-99434) and anti p-GSK-3β (Tyr216) (1:1000, Catalog No. # 44-604G) (ThermoFisher Scientific Inc., USA), Afterward, membranes were washed and then incubated with horseradish peroxidase-conjugated secondary antibody anti-rat immunoglobulin (1:2000; Fluka, St. Louis, MO, USA). Finally, the blots were developed with enhanced chemiluminescence detection reagents (Amersham Biosciences, Arlington Heights, IL, USA). The amount of CREB, AKT, mTOR, and GSK-3β proteins was quantified by densitometric analysis using a scanning laser densitometer (GS-800 system, Bio-Rad, Hercules, CA, USA). Finally, chemiluminescence detection was performed with the Amersham detection kit according to the manufacturer’s protocols and exposed to X-ray film. The protein bands intensities were  quantified by densitometric analysis of the autoradiograms using a scanning laser densitometer (Biomed Instrument Inc., USA). Results were expressed as arbitrary units after normalization with β-actin protein expression (Catalog No. **#**MA5-15739) (ThermoFisher Scientific Inc., USA).

### Quantitative real-time PCR analysis

Striatal TrKB and forkhead box (FoxO1) mRNA expressions were assessed using qRT-PCR technique. Total RNA was extracted from striatal tissue using SV Total RNA Isolation system (Promega, Madison, WI, USA) and the purity of obtained RNA was verified spectrophotometrically at OD 260/280 nm. The extracted RNA was then reverse transcribed into complementary DNA using RT-PCR kit (Stratagene, La Jolla, CA, USA) according to the manufacturer's procedure. QRT-PCR was performed using SYBR Green JumpStart Taq ReadyMix (Sigma-Aldrich, St. Louis, MO, USA) as described by the manufacturer. The primers were obtained from Macrogen Inc. Seoul, South Korea and were designed using Primer-Blast (Basic Local Alignment Search Tool) of NCBI (National Center for Biotechnology Information). The primer sequences are listed in Table 1. Briefly, in a 25 μl reaction volume, 5 μl of complementary DNA was added to 12.5 μl SYBR Green mixture, 5.5 μl RNase free water, and 2 μl of each primer (5 pmol/μl). The PCR amplifications were performed with the following steps: initial denaturation at 50 °C for 2 min followed by 40 cycles of denaturation at 95 °C for 15 s, annealing at 60 °C for 1 min, and extension for 60 s at 72 °C. After the qRT-PCR run, the relative expression of the target gene was obtained using the 2^−ΔΔCT^ formula using β-actin as a housekeeping gene (Pfaffl 2001).

**Table 1:** The primer sequences used in RT-PCR

Gene	Accession no.	Forward primer	Reverse primer
TrKB	NM_012731.3	5′-CTACCTGGCATCCCAACACT- 3′	5′-CTCGGTGGTGAATTTCCTGT-3′
FoxO1	NM_001191846.3	5′-CCGACCTCATCACCAAGG-3′	5′-TCT CCAGGACCCTCTTGC-3′
β-Actin	XM_039089807.1	5′-CGTTGACATCCGTAAAGACCTC-3′	5′-TAGGAGCCAGGGCAGTAATCT-3′

## Histopathological examination

### H&E stain

Brains were fixed in 10% formalin for 24 h. The specimens were dehydrated in ascending grades of alcohol, cleared in xylene, and embedded in paraffin at 56 degrees in a hot air oven for 24 h. Paraffin beeswax tissue blocks were prepared for sectioning at 4 μm thickness by a sledge microtome. The obtained tissue sections were collected on glass slides, deparaffinized, stained by hematoxylin and eosin (H&E) stain, and examined through the light electric microscope. During the histopathological analysis, the investigator was blinded to sample identity, and sample coding and decoding were performed by an independent experimenter.

### Immunohistochemistry

The immunohistochemical technique was used to assess striatal dopaminergic tyrosine hydroxylase. The brain samples were processed into paraffin blocks; thereafter, 4 μm sections were prepared on positively charged glass slides. Endogenous peroxidase activity was quenched by first incubating the specimens in 3% hydrogen peroxide. The specimens were then incubated with primary monoclonal anti-tyrosine hydroxylase (TH) antibody obtained from Abcam, USA (Cat. # ab112), followed by sequential incubations with biotinylated link antibody and peroxidase-labeled streptavidin (Dako, Carpinteria, CA, USA). Labeling was then revealed by diaminobenzidine chromogen. Slides were counterstained with hematoxylin, dehydrated, covered and examined through the light electric microscope (Olympus CX21, Tokyo, Japan). The area percent of TH-positive fibers in the striatum was determined microscopically (magnification × 40) using the Leica Qwin 500 Image Analyzer (Leica Microsystems, Wetzlar, Germany) from four randomly selected fields for each animal. The results were presented as the area percentage of TH-positive cell.

### Statistical analysis

Data sets are presented as mean ± S.E.M. Comparison between groups was carried out using one-way analysis of variance (ANOVA), followed by Tukey’s multiple comparisons test; except the histopathological scoring data were analyzed using Kruskal–Wallis nonparametric one-way ANOVA followed by Dunn’s multiple comparisons test and presented as median and range. A probability level of less than 0.05 was accepted as statistically significant. Statistical analysis was performed using GraphPad Prism software version 6 (San Diego, CA, USA).

## Results

### Roflumilast ameliorates rotenone-induced behavioral changes in rats

**Fig. 2:**
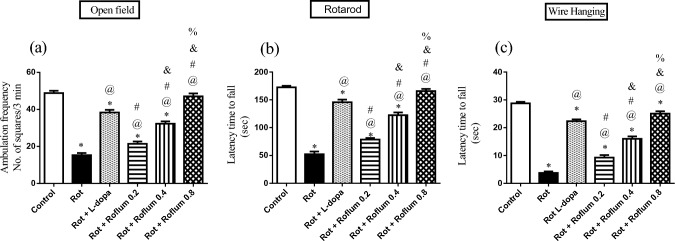
Roflumilast ameliorates rotenone-induced behavioral changes in rats. **a** Ambulation frequency, **b** rotarod, and **c** wire hanging. Each bar with vertical line represents the mean ± S.E.M. (*n* = 12–15). *Significantly different from control, ^@^significantly different from rotenone, ^#^significantly different from L-dopa, ^&^significantly different from Roflum 0.2 mg/kg, ^%^significantly different from Roflum 0.4 mg/kg. Statistical analysis was performed using one-way ANOVA followed by Tukey–Kramer multiple comparison test at *p* < 0.05. *Rot* rotenone, *Roflum* roflumilast

Rats receiving rotenone showed worsened locomotor activity as manifested by significantly decreased ambulation frequency, deteriorated motor coordination in rotarod mobility, and significantly lessened latency time to fall from the wire as compared to the control group (Fig. 2). L-dopa alleviated rotenone-induced motor disability, where ambulation frequency, mobility time on rotarod, and the latency time to fall from the wire were boosted by 2.5-fold. 2.8-fold, and 6-fold, respectively, as compared to rotenone group. Treatment with roflumilast (0.2, 0.4, or 0.8 mg/kg) reversed rotenone’s injurious effects via amplifying ambulation frequency by 1.4-fold, 2-fold, and 3-fold as well as enhancing mobility time in rotarod test by 1.5-fold, 2.3-fold, and 3.2-fold, respectively as compared with the rotenone group (Fig. 2a and b). Moreover, roflumilast (0.2, 0.4, and 0.8 mg/kg) succeeded to significantly increase the latency time to fall from the wire by 2.5-fold, 4.3-fold, and 6.7-fold, respectively (Fig. 2c) as compared with the rotenone group.

### Roflumilast amends rotenone-induced alterations in striatal cAMP, PI3K, and AKT in rats

**Fig. 3:**
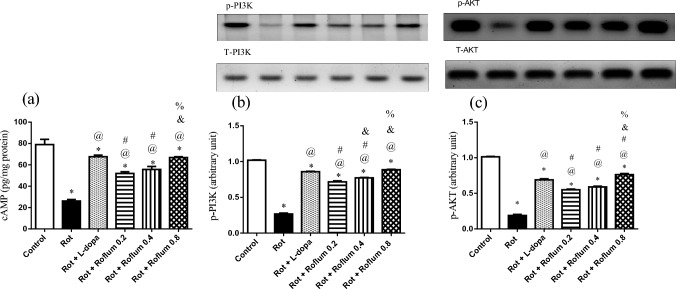
Roflumilast amends rotenone-induced alterations in striatal contents of **a** cAMP, **b** PI3K, and **c** AKT in rats. Each bar with vertical line represents the mean ± S.E.M. (*n* = 6). *Significantly different from control, ^@^significantly different from rotenone, ^#^significantly different from L-dopa, ^&^significantly different from Roflum 0.2 mg/kg, ^%^significantly different from Roflum 0.4 mg/kg. Statistical analysis was performed using one-way ANOVA followed by Tukey–Kramer multiple comparison test at *p* < 0.05. *Rot* rotenone, *Roflum* roflumilast

Striatal tissues of rotenone-treated rats revealed a dramatic decline in cAMP level, PI3K, and AKT expressions by 67%, 74%, and 81%, respectively, as compared to control group values (Fig. 3). Treatment with L-dopa resulted in a significant elevation of cAMP level (2.6-fold), PI3K expression (3.2-fold), and AKT expression (3.6-fold) as compared to rotenone group. Similarly, roflumilast (0.2, 0.4, or 0.8 mg/kg) elicited a remarkable upsurge in cAMP level by 1.9-fold, 2.1-fold, and 2.6-fold, respectively, which triggered massive increments in PI3K expression by 2.7-fold, 2.9-fold, and 3.3-fold, respectively, and AKT expression by 2.9-fold, 3.1-fold, and 4-fold, respectively, as compared to rotenone group values.

### Roflumilast reverses rotenone-induced alterations of striatal CREB, BDNF and TrKB in rats

**Fig. 4:**
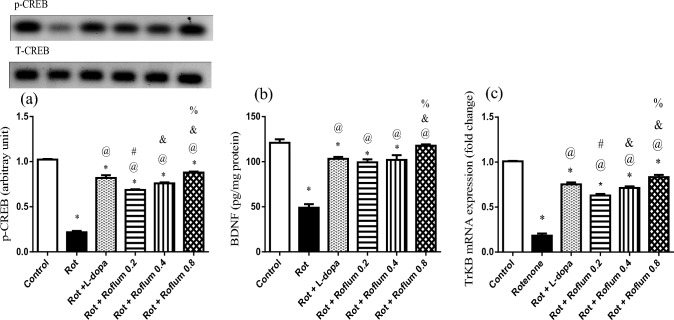
Roflumilast reverses rotenone-induced alterations of striatal **a** CREB, **b** BDNF, and **c** TrKB in rats. Each bar with vertical line represents the mean ± S.E.M (*n* = 6). *Significantly different from control, ^@^significantly different from rotenone, ^#^significantly different from L-dopa, ^&^significantly different from Roflum 0.2 mg/kg, ^%^significantly different from Roflum 0.4 mg/kg. Statistical analysis was performed using one-way ANOVA followed by Tukey–Kramer multiple comparison test at *p* < 0.05. *Rot* rotenone, *Roflum* roflumilast

Striatal tissues of rotenone-treated rats showed a marked depletion in p-CREB expression, BDNF level, and TrKB mRNA expression by 79%, 60%, and 82%, respectively, as compared to control group values (Fig. 4). Treatment with L-dopa resulted in a significant elevation of p-CREB expression (3.8-fold) as compared to rotenone group. Similarly, roflumilast (0.2, 0.4, or 0.8 mg/kg) escalated p-CREB expression by 3.1-fold, 3.5-fold, and 4.1-fold, respectively, as compared to rotenone group. In parallel, BDNF level and TrKB mRNA expression were highly augmented by L-dopa and roflumilast. Distinctly, roflumilast (0.8 mg/kg) was the most effective in boosting the prosurvival BDNF cascade through triggering magnificent increments of BDNF level (2.4-fold) as compared to the rotenone group (Fig. 4b). This effect was verified by significant concurrent amplifications in the mRNA expression of the downstream BDNF effector TrkB (4.5-fold) as compared to the rotenone group (Fig. 4c).

### Roflumilast alleviates rotenone-induced alterations in striatal contents of SIRT1, IGF1, and PTP1B in rats

**Fig. 5:**
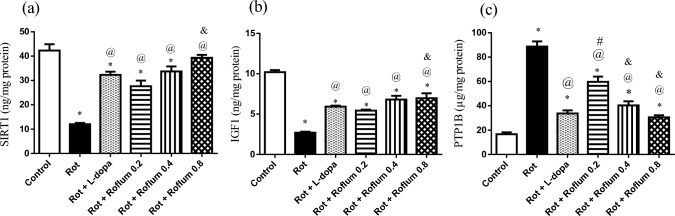
Roflumilast alleviates rotenone-induced alterations in striatal contents of **a** SIRT1, **b** IGF1, and **c** PTP1B in rats. Each bar with vertical line represents the mean ± S.E.M. (*n* = 6). *Significantly different from control, ^@^significantly different from rotenone, ^#^significantly different from L-dopa, ^&^significantly different from Roflum 0.2 mg/kg, ^%^significantly different from Roflum 0.4 mg/kg. Statistical analysis was performed using one-way ANOVA followed by Tukey–Kramer multiple comparison test at *p* < 0.05. *Rot* rotenone, *Roflum* roflumilast

Striatal SIRT1 and IGF1 levels were reduced after rotenone injection by 72% and 74%, respectively, as compared to their normal control counterparts. These effects were mitigated by treatment with L-dopa and roflumilast (0.2, 0.4, or 0.8 mg/kg) that boosted striatal SIRT1 level by 2.7-fold, 2.3-fold, 2.8-fold, and 3.3-fold, respectively, and striatal IGF1 level by 2.2-fold, 2-fold, 2.5-fold, and 2.6-fold, respectively, as compared to the rotenone group (Fig. 5). Besides, L-dopa and roflumilast (0.2, 0.4, or 0.8 mg/kg) significantly suppressed PTP1B, a major IGF1 inhibitor, level by 62%, 32%, 55%, and 66%, respectively, in comparison to the rotenone group.

### Roflumilast attenuates rotenone-induced alterations of striatal mTOR, Nrf2, Gsk-3β, NF-κB, FoxO1, and caspase-3 in rats

**Fig. 6:**
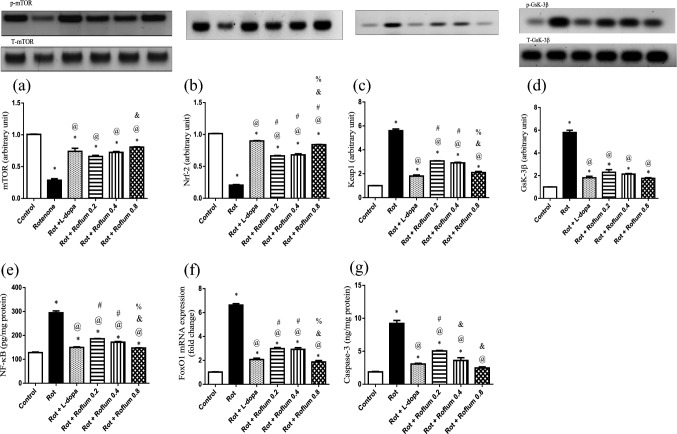
Roflumilast attenuates rotenone-induced alterations of striatal **a** mTOR, **b** Nrf2, **c** Keap1, **d** GSK-3β, **e** NF-κB, **f** FoxO1, and **g** caspase-3 in rats. Each bar with vertical line represents the mean ± S.E.M. (*n* = 6). *Significantly different from control, ^@^significantly different from rotenone, ^#^significantly different from L-dopa, ^&^significantly different from Roflum 0.2 mg/kg, ^%^significantly different from Roflum 0.4 mg/kg. Statistical analysis was performed using one-way ANOVA followed by Tukey–Kramer multiple comparison test at *p* < 0.05. *Rot* rotenone, *Roflum* roflumilast

Repeated rotenone injection caused an obvious 72% decrease in striatal mTOR expression, along with 80% decrement in Nrf2 level as compared to the control group. Rotenone’s deleterious effect extends to exhibit a prominent increase in Keap1 expression by 5.6-fold, in addition to escalating inflammatory and apoptotic mediators including Gsk-3β expression, NF-κB level, FoxO1 mRNA expression, and caspase-3 level by 5.7-fold, 2.3-fold, 6.4-fold, and 4.9-fold, respectively, as compared to control rats (Fig. 6). On the contrary, L-dopa and roflumilast (0.2, 0.4, or 0.8 mg/kg) markedly enhanced neurogenesis which was evidenced by upregulating striatal mTOR expression by 2.5-fold, 2.3-fold, 2.5-fold, and 2.8-fold along with upleveling Nrf2 level by 4.3-fold, 3.2-fold, 3.3-fold, and 4.1-fold, respectively. Moreover, treatment with L-dopa and roflumilast (0.2, 0.4 or 0.8 mg/kg) dampens Keap1 expression by 68%, 45%, 48%, and 63%, respectively, and switched “off” the surge of Gsk-3β expression by 69%, 60%, 63%, and 70%, respectively, NF-κB level by 49%, 37%, 42%, and 50%, respectively, FoxO1 mRNA expression by 69%, 55%, 56%, and 72%, respectively, and caspase-3 level by 67%, 45%, 61%, and 73%, respectively, versus the rotenone-treated group values.

### Roflumilast mitigates rotenone-induced histopathological changes

**Fig. 7:**
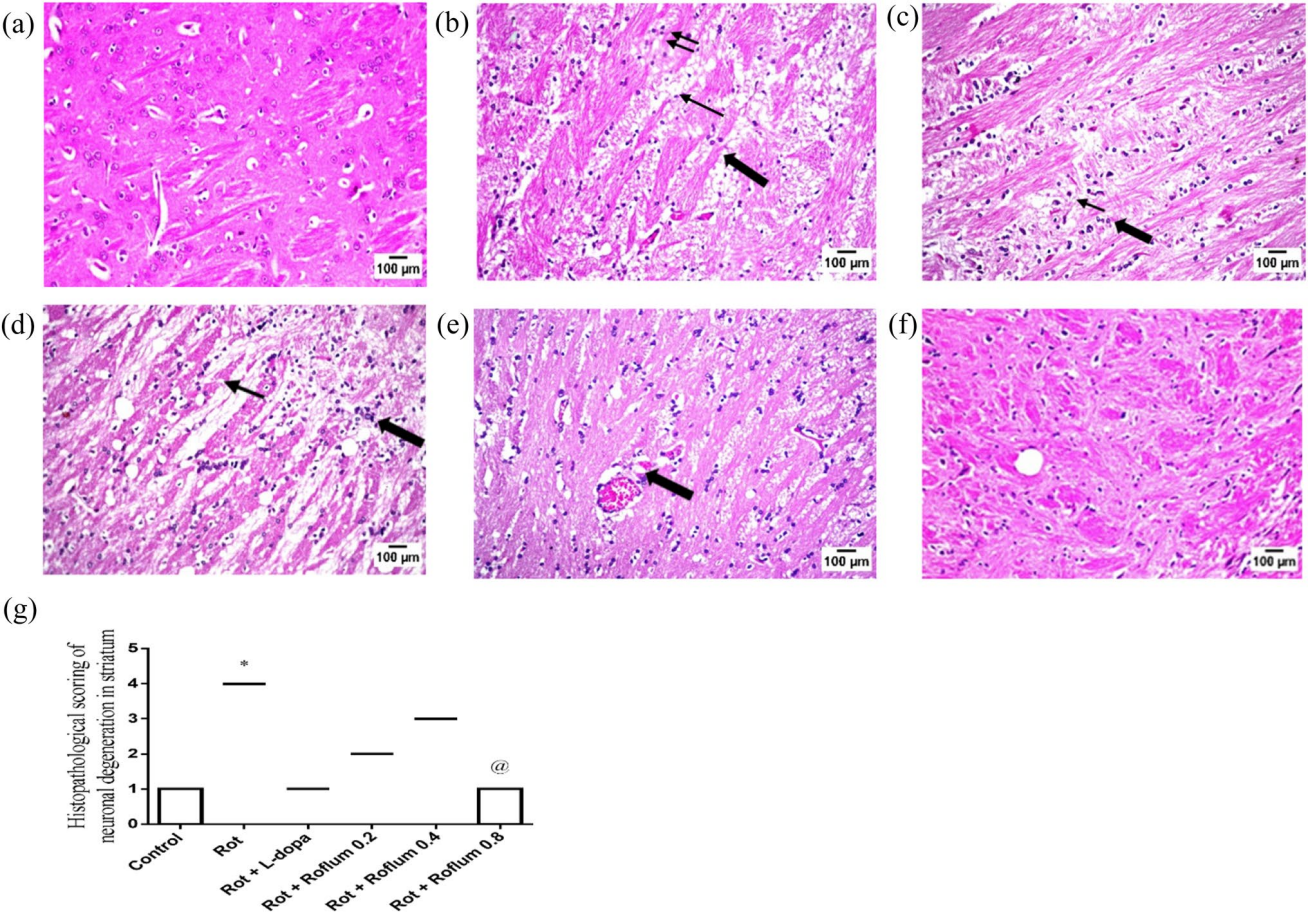
Roflumilast mitigates rotenone-induced histopathological changes. Striatal sections from control rats stained with hematoxylin and eosin (H&E). Striatal tissue in control group showed normal histological structure (**A**), while rats receiving rotenone showed encephalomalacia (one arrow), nuclear pyknosis (two arrows), degenerative gliosis (thick arrow) of several striatal neurons (**B**). Striatal sections from rats treated with L-dopa showed slight gliosis (**C**). Striatal sections from rats treated with roflumilast (0.2 or 0.4 mg/kg) showed moderate gliosis with dispersion of encephalomalacia (**D** and **E**, respectively). Sections from rats treated with roflumilast (0.8 mg/kg) showed almost manifested intact nigral neurons with visible nuclei (**F**). Histological scoring of neuronal degeneration in the striatum (**G**). Data are expressed as median and range of three rats per group; *versus control, ^@^versus Rot. Statistical analysis was done by Kruskal–Wallis one-way ANOVA followed by Dunns multiple comparison test at *p* < 0.05

Sections from control group showed normal histological striatal neuronal structure (Fig. 7A). On the contrary, striata of rotenone-treated rats revealed severe focal encephalomalacia associated with neuronal degeneration, dark pyknotic nuclei accompanied by severe diffused gliosis (Fig. 7B). These noxious histopathological changes were reversed in the striata of parkinsonian rats treated with L-dopa and roflumilast (0.2 or 0.4 mg/kg), where fewer scattered degenerated neurons and mild interspersed gliosis were seen (Fig. 7C, D, and E). Rats treated with roflumilast (0.8 mg/kg) revealed marked improvement with almost normal striatal architecture, intact neurons, and very slight gliosis (Fig. 7F).

### Roflumilast restores TH enzyme immunoreactivity

**Fig. 8:**
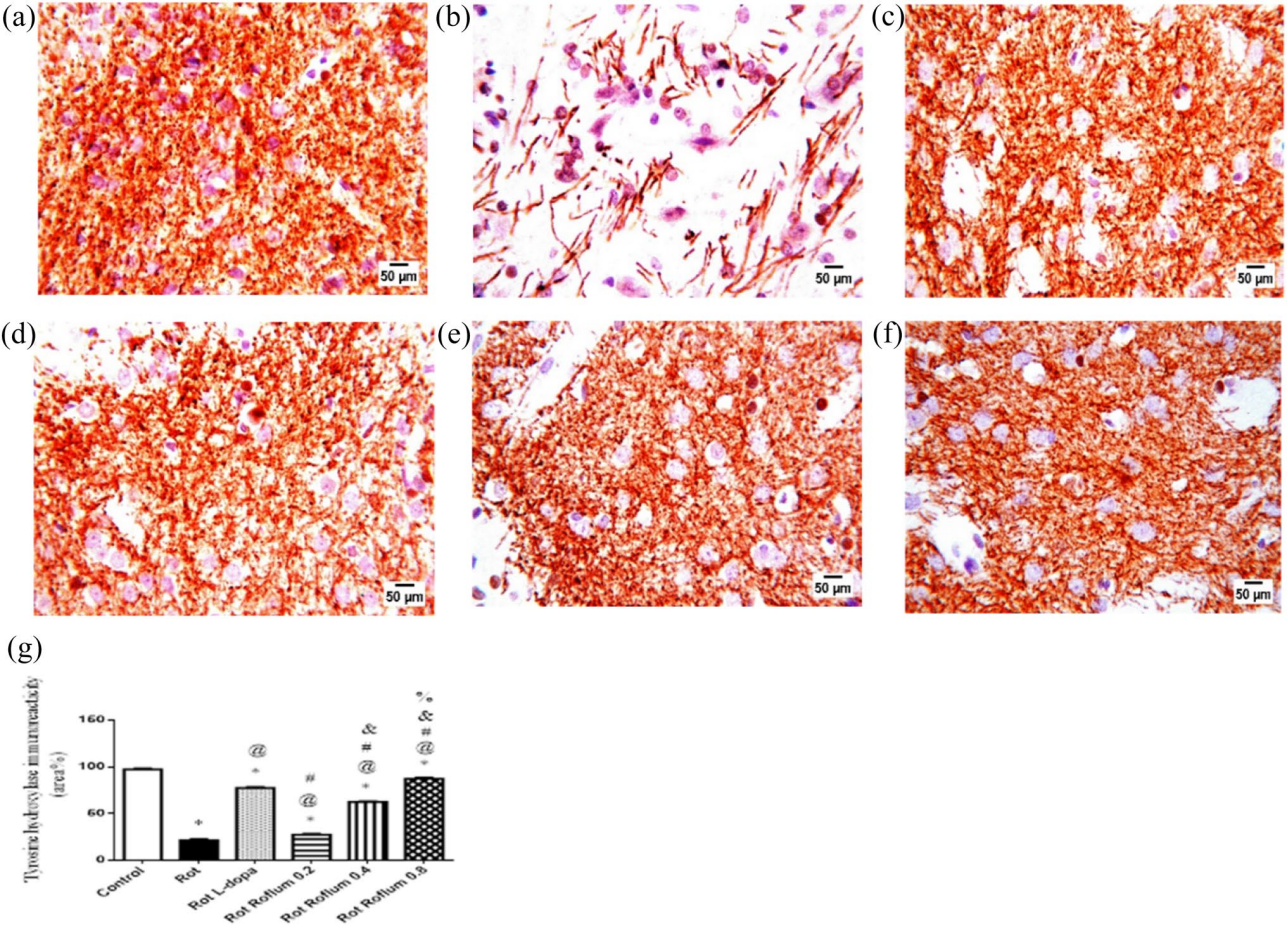
Roflumilast restores TH enzyme immunoreactivity. Immunohistochemical expression of TH in control rats (**A**) showed marked strong tyrosine hydroxylase expression, while rats receiving rotenone (**B**) showed very weak tyrosine hydroxylase expression. Sections from rats treated with L-dopa showed moderate tyrosine hydroxylase expression (**C**). Sections from rats treated with roflumilast (0.2 or 0.4 mg/kg) showed moderate tyrosine hydroxylase expression (**D** and **E**, respectively). Sections from rats treated with roflumilast (0.8 mg/kg) showed an obvious tyrosine hydroxylase expression (**F**). **G**: The area % of TH-immunoreactivity. Data were expressed as mean and range of three rats per group; *versus control, ^@^versus Rot, ^#^versus L-dopa, ^&^versus Roflum 0.2 mg/kg, ^%^versus Roflum 0.4 mg/kg. Statistical analysis was performed using one-way ANOVA followed by Tukey–Kramer multiple comparison test at *p* < 0.05. *Rot* rotenone, *Roflum* roflumilast

Figure 8 revealed a prevalent injury of striatal dopaminergic neuronal fibers in rotenone-treated rats, declared by a significant reduction (85%) of TH immunoreactivity as compared to the control group. Administration of roflumilast (0.2 or 0.4 mg/kg) alleviated rotenone-induced dopaminergic degeneration as shown by mild increased striatal TH immunoreactivity. Interestingly, roflumilast (0.8 mg/kg) outperformed L-dopa in reversing the reduced TH immunoreactivity in the rotenone model displaying fourfold increase in striatal TH immunoreactive positive cells in roflumilast (0.8 mg/kg)-treated group versus 3.6-fold increase in L-dopa-treated group.

## Discussion

The present study reveals the neuroprotective effect of roflumilast against rotenone-induced PD in rats. This notion is supported by: (a) an improvement in rats’ motor activity and coordination; (b) the preservation of dopaminergic neurons in the striatum; (c) an increase in cAMP level; (d) the activation of PI3K/AKT axis; (e) the stimulation of CREB/BDNF/TrKB and SIRT1/PTP1B/IGF1 signaling cascades; (f) an upsurge in the prosurvival proteins mTOR and Nrf2; (g) the anti-inflammatory activity via halting GSK-3β and NF-ĸB; and (h) the suppression of apoptotic markers FoxO1 and caspase-3.

Chronic rotenone exposure in rats elicits neuropathological and behavioral features mimicking the gradual progression of PD observed in humans (Xiong et al. 2012; Johnson and Bobrovskaya 2015). In line, repeated exposure of rats to rotenone in the current study resulted in dopaminergic neuronal death and reduced locomotor activity, loss of grip strength, and a decrease in the fall time in the rotarod test, indicating motor impairments accompanied with severe loss of dopaminergic neurons in the SN, which are in line with previous studies (Abdelkader et al. 2020; El-Saiy et al. 2022; El-Latif et al. 2023).

Empirical evidence revealed the potential role of defective PI3K/AKT signaling in neurodegenerative disorders including PD (Malagelada et al. 2008; Levy et al. 2009; Goyal et al. 2023). Additionally, various drugs were reported to have neuroprotective effects in PD via PI3K/AKT activation (Hu et al. 2018; Huang et al. 2021; Shao et al. 2022). AKT is a master signaling serine/threonine-specific protein kinase found downstream of PI3K, possessing a greatly expanded functional repertoire as it maintains cell growth and proliferation by controlling the phosphorylation of a vast array of trafficking nodes (Long et al. 2021). Activation and phosphorylation of PI3K/AKT signaling are initiated after the binding of diverse convergent neurotrophic factors, cytokines and insulin to their receptors (Rai et al. 2019; Radak et al. 2020; Lu et al. 2023b). PI3K/AKT signaling can be activated via cAMP/PKA-dependent pathways (Wang and Liu 2023). Activated cAMP/PKA pathway in response to PDE4 inhibition was reported to ameliorate movement deficits accompanied with PD and to preserve the survival of TH-positive neurons in the SN (Yang et al. 2008; Erro et al. 2021). In line, our results revealed that roflumilast enhanced cAMP level with subsequent activation of PI3K and AKT. The elevation of cAMP level by roflumilast is attributed to its ability to inhibit PDE4. These findings are in harmony with a recent study demonstrating the potential neuroprotective effect of roflumilast against rotenone-induced PD in rats through the activation of the cAMP/PKA pathway (Essam and Kandil 2023).

Activated cAMP/PKA signaling phosphorylates CREB at Ser133 and promotes its transcriptional activity (Guo et al. 2017). CREB activation is vital for neuronal survival, synaptic transmission and transcription of antioxidant genes (Lin et al. 2015; Wu et al. 2018). CREB phosphorylation at ser 133 is associated with increased expression of NURR1, one of the essential genes crucial for nigral dopaminergic neurons survival (Xu et al. 2022). Activated p-CREB evokes the transcription of BDNF (Narasimhamurthy et al. 2022), which is a prosurvival neurotrophic factor that is highly expressed in the striatum (Palasz et al. 2020). BDNF elicits its neuroprotective action after binding to TrKB receptor and its phosphorylation (Jin 2020). BDNF/TrKB axis promotes neurogenesis by stimulating AKT pathway (Bai et al. 2019). In the present study, roflumilast increased p-CREB expression, BDNF level, and TrKB mRNA expression in the striatum of rotenone-treated rats. These findings reveal the potential role of activating p-CREB/BDNF/TrKB signaling in the neuroprotective effect of roflumilast observed in the current study via stimulation of PI3K/AKT axis. In parallel, previous studies showed that activation of p-CREB/BDNF/TrKB signaling pathway was neurorestorative in PD animal models through induction of PI3K/AKT signaling cascade (Yu et al. 2019; Mousa et al. 2023; Singh et al. 2023).

cAMP/PKA activation could also trigger PI3K/AKT signaling and exhibit neuroprotective properties in neurodegenerative disorders via SIRT1 downstream cascade (Wang et al. 2020b), which is a nicotinamide adenine dinucleotide (NAD)-dependent histone deacetylase (Ichiro and Johnson 2018). Modulating SIRT1 activity counteracts the aging process, inducing neuronal vitality, adjusting cellular homeostatic defense against stress, and superimposing a paramount life longevity impact (Mishra et al. 2021; Ziętara et al. 2022). SIRT1 overexpression also fosters insulin signaling by impairing PTP1B, a major inhibitor of insulin receptor (Sun et al. 2007; Lu et al. 2023a; Wu et al. 2023). In response to PTP1B inhibition, IGF1 binds to its corresponding receptor and triggers downstream neuroprotective signaling pathways of PI3K/AKT (Yang et al. 2018; Li et al. 2023a; Tuohongerbieke et al. 2023). α-Syn aggregation in PD could be suppressed via augmenting SIRT1 and IGF1 activities (Kakoty et al. 2023). Herein, we showed that roflumilast increased levels of SIRT1 and IGF1 along with reduced expression of PTP1B in striatum of rotenone-injected rat, verifying the role of this signaling in the neuroprotective effect of roflumilast. In agreement, roflumilast was showed to abolish doxorubicin-induced inflammation and diabetes-associated cardiac dysfunction by upregulating SIRT1 (Zhang et al. 2021).

The crosstalk between CREB/BDNF/TrkB and SIRT1/PTP1B/IGF1 signaling pathways offered by roflumilast leads to the activation of PI3K/AKT trajectory. Active PI3K/AKT signaling was manifested to evoke neuroprotection in PD via upregulating numerous downstream prosurvival substrates, including mTOR (Zheng et al. 2021a) and Nrf2 (Liu et al. 2021). Moreover, PI3K/AKT signaling axis was demonstrated to alleviate neuroinflammation and apoptosis in PD via downregulating GSK-3β, NF-ĸB, FoxO1 (Cheong et al. 2020) and caspase-3 (Feng and Xi 2022).

mTOR is a master regulator kinase that possesses a crucial role in cellular development, neuronal survival, and synaptic plasticity (Querfurth and Lee 2021). Overexpression of mTOR is a prerequisite for neuronal viability in vitro PD models (Gugliandolo et al. 2020; El-Sherbeeny et al. 2020). The PI3K/AKT/mTOR signaling network of the nervous system governs neuronal differentiation and survival, along with learning, memory, synaptic plasticity, and neuronal oxidative stress (Li et al. 2020). Our results herein showed that roflumilast prohibits rotenone neurotoxicity by elevating p-mTOR protein expression.

PI3K/AKT signaling was elucidated to also promote the activation of Nrf2 signaling in Alzheimer’s disease (Lin et al. 2022) and traumatic brain injury (Cheng et al. 2023). Nrf2 regulates transcription of plenty of genes encoding protective molecules against inflammation and oxidative stress (Singh et al. 2010; Li et al. 2014). Under normal physiological conditions, Nrf2 is chelated by its endogenous inhibitor, kelch-like ECH-associated protein 1 (Keap1) within the cytoplasm (Luo et al. 2021). Nrf2 is activated after being liberated from Keap1-mediated degradation and gets imported to the nucleus (Chen et al. 2022). Upon nuclear translocation, Nrf2 stimulates the transcription of multiple antioxidant and anti-inflammatory genes after binding to antioxidant response elements (ARE) located in the promoter region of those genes (Magesh et al. 2012). Thus, Nrf2 activators could be effective in breaking down α-syn aggregates and hampering NF-κB-associated neuroinflammation elucidated in PD via maintaining cellular redox homeostasis (Chakkittukandiyil et al. 2022; de Siqueira et al. 2023). Our results clarified that roflumilast ameliorates rotenone-induced neurotoxicity by upsurging striatal Nrf2 expression along with downregulating Keap 1 expression, which is in line with a previous study showing the neuroprotective effect of roflumilast against ischemic stroke-induced neuronal damage via activating Nrf2 (Xu et al. 2021).

PI3K/Akt pathway also provokes a prosurvival effect by inhibiting glycogen synthase kinase 3 β (GSK-3β), which is reported to be activated in PD (Teixeira et al. 2016; Arab et al. 2021). Consequences of GSK-3β activation not only encompass increasing α-synuclein aggregates in vitro PD models (Arciniegas Ruiz and Eldar-Finkelman 2022) but also extend to amplification of mitochondrial apoptosis (King et al. 2001; Linseman et al. 2004). GSK-3β participates in neuroinflammatory progression through activating the NF-κB pathway which exacerbates inflammatory insults in PD (Huang et al. 2018; Samim Khan et al. 2023). PI3K/AKT signaling favorable impact extends to halting the transcription of FoxO1-driven pro-apoptotic genes (Sánchez-Alegría et al. 2018; Maiese 2021). FoxO1 negatively regulates TH expression, the rate-limiting enzyme of dopamine synthesis (Doan et al. 2016). Our results showed that treatment with roflumilast antagonized rotenone-induced neurotoxicity through downregulating GSK-3β, NF-κB, FoxO1 and caspase-3. These findings are in harmony with previous studies emphasizing the same fruitful impact of PDE4 inhibition in suppressing those injurious parameters in other degenerative models (Wang et al. 2020b; Arcaro et al. 2021; Hasan et al. 2022). Our study revealed that roflumilast exerted neuroprotective effects in rotenone-induced neurotoxicity in rats. These neuroprotective effects were mediated via the crosstalk between CREB/BDNF/Trk Bans SIRT1/PTP1B/IGF1 signaling pathways which activates PI3K/AKT trajectory along with the anti-inflammatory and anti-apoptotic effects. Therefore, PDE4 inhibition by roflumilast is likely to offer a reliable persuasive avenue in curing PD via PI3K/AKT signaling activation.

## Data Availability

Data will be available upon request.

## Abbreviations

PD: Parkinson’s disease
AKT: Protein kinase B
BDNF: Brain-derived neurotrophic factor
SIRT1: Silent information regulator type 1
PI3K: Phosphoinositide 3-kinase
cAMP: Cyclic adenosine monophosphate
TrKB: Tropomyosin receptor kinase B
IGF1: Insulin growth factor 1
